# Measurement properties of the SARC-T: test-retest reliability, agreement and minimal detectable change in older adults with sarcopenia

**DOI:** 10.3389/fragi.2026.1822206

**Published:** 2026-05-29

**Authors:** Blanca Pedauyé-Rueda, José Luis Maté-Muñoz, Juan Hernández-Lougedo, Cristian Solís-Mencía, Rebeca Bueno-Fermoso, Arturo Cano-Uceda, Cristina Ojedo-Martín, Pablo García-Fernández

**Affiliations:** 1 HM Faculty of Health Sciences, Camilo José Cela University, Madrid, Spain; 2 HM Hospitals Health Research Institute, Madrid, Spain; 3 Department of Physiotherapy, Complutense University of Madrid, Madrid, Spain; 4 Department of Medicine, Faculty of Health Sciences, University of Deusto, Bilbao, Bizkaia, Spain; 5 Department of Nursing, Complutense University of Madrid, Madrid, Spain; 6 Faculty of Health Sciences, Alfonso X El Sabio University, Madrid, Spain

**Keywords:** aging, assessment, reliability, sarcopenia, SARC-T

## Abstract

**Background:**

The Sarcopenia Optoelectronic Chair-Rise Velocity Test (SARC-T) has been proposed as an instrumented chair-rise measure related to physical function in sarcopenia; however, its test-retest measurement properties have not been established. This study evaluated the test-retest reliability, agreement, measurement error and minimal detectable change at the 95% confidence level (MDC95) of the SARC-T in older adults with sarcopenia.

**Methods:**

An observational methodological test-retest study was conducted in 55 institutionalized older adults with sarcopenia diagnosed according to EWGSOP2 criteria. The SARC-T was assessed in two sessions separated by 5 days under standardized conditions. The primary outcome was the best mean propulsive velocity recorded during the concentric phase. Test-retest reliability was assessed using ICC (3,1), Lin’s concordance correlation coefficient (CCC), standard error of measurement (SEM), MDC95, Bland–Altman analysis, and a linear mixed-effects model.

**Results:**

Test-retest reliability was high (ICC = 0.93, 95% CI 0.88–0.96; CCC = 0.93, 95% CI 0.86–0.96). Measurement error was low (SEM = 0.017 m/s), and MDC95 was 0.05 m/s. Bland–Altman analysis showed a small but statistically significant mean bias of +0.008 m/s (p = 0.015), with 95% limits of agreement from −0.042 to +0.058 m/s, and additional analysis showed statistically significant proportional bias (slope = 0.161; p = 0.009). A small systematic session effect was also observed in the mixed-effects model (β = +0.008 m/s; 95% CI 0.002–0.018; p = 0.012).

**Conclusion:**

Under the standardized conditions of the present protocol, the SARC-T showed high test-retest reliability and low measurement error; however, the presence of statistically significant mean and proportional bias indicates that agreement is not fully uniform across the measurement range and should be interpreted with caution, particularly at the individual level. The MDC95 may help interpret whether longitudinal changes in mean propulsive velocity are likely to exceed measurement-related fluctuation. These findings should be considered within the context of this specific population of institutionalized older adults with sarcopenia.

## 1 Introduction

Sarcopenia is an age-related disease characterized by the progressive decline in skeletal muscle mass and function and is associated with adverse health outcomes (Mayhew et al., 2019; Kirk et al., 2024). In 2016, the World Health Organization added it to the International Classification of Diseases and Related Health Problems under code ICD-10-CM M62.84 (Anker et al., 2016). Despite the high healthcare burden it generates, it remains underdiagnosed in many cases as a result of the use of different classifications and cut-off points (Janssen et al., 2002; Scott et al., 2014; Petermann-Rocha et al., 2022), which is particularly relevant in healthcare settings where frailty, falls, and loss of autonomy directly influence the need for care and the use of healthcare resources (Gariballa and Alessa, 2013; Kim et al., 2014; WHO, 2019; Giglio et al., 2018; Cruz-Jentoft and Sayer, 2019).

In clinical practice, identifying sarcopenia requires tools that are not only valid but also feasible in clinics and healthcare units. The consensus developed by the European Working Group on Sarcopenia in Older People 2 (EWGSOP2) proposes a stepwise approach to diagnosis, with screening beginning with the Strength, Assistance with walking, Rise from a chair, Climb stairs, and Falls (SARC-F) questionnaire. Sarcopenia is considered probable in cases of low muscle strength assessed by handgrip strength and/or the 5-sit-to-stand test (5-STST). The diagnosis is confirmed by evidence of low muscle quantity or quality, and severity is established based on physical performance, usually through a walking speed test (Cruz-Jentoft et al., 2019). However, the routine application of this algorithm may be limited by logistical and clinical barriers. The availability of equipment to measure strength with calibrated dynamometry and to quantify muscle quantity/quality, together with limited care times and the need for space to perform walking tests, can hinder its application in routine practice. In addition, patient tolerance is not always optimal, as comorbidities, pain, fatigue, or fear of falling often coexist, complicating the standardization of the procedure and its systematic implementation in real-world settings.

Among functional tests, the 5-STST is commonly used to assess lower limb function, and its performance is associated with frailty, disability, and poorer quality of life (Bohannon et al., 2010). It is also widely used in neuromuscular and musculoskeletal disorders (Muñoz-Bermejo et al., 2021; Polidori et al., 2024). However, its applicability may be reduced in frail populations or those with functional limitations, as a significant proportion of older adults are unable to complete five consecutive repetitions (Bohannon, 2011). At the same time, in scenarios with limited consultation times, having brief and easily standardizable procedures is particularly relevant (Beaudart et al., 2019). In this context, an alternative based on the clinically relevant gesture of getting up from a chair, but with fewer repetitions required and greater practicality for use in healthcare, is of interest.

The Sarcopenia Optoelectronic Chair-Rise Velocity Test (SARC-T) has recently been proposed as an instrumented chair-rise test that quantifies average propulsive velocity at which a subject rises from a chair, as an adjunct test to assess the probability and severity of sarcopenia. The test consists of standing up from a chair as quickly as possible and sitting down at a controlled velocity, without arm assistance, and recording the average propulsive velocity during the concentric phase of the movement. This is performed twice, and the maximum value obtained is recorded, given the high intra-session reliability and consistency between repetitions observed in both clinical populations and healthy adults. A previous study of the SARC-T provided evidence of concurrent validity by associating it with functional tests of the framework and discriminant validity by differentiating between older adults with sarcopenia and controls without sarcopenia. Furthermore, no significant changes were observed in physiological variables between the pre- and post-test assessments, which supports the feasibility of the procedure in older adults with and without sarcopenia. In addition, the study provided evidence of moderate-to-high associations with the functional tests recommended by the EWGSOP2 framework for the assessment of muscle strength and physical performance. Taken together, these findings suggest that the SARC-T could represent a complementary performance-based measure for the assessment of physical function related to sarcopenia, particularly in settings where other tests may be less feasible or where associated comorbidities may influence performance (Pedauyé-Rueda et al., 2026). However, for use in clinical follow-up and decision-making, robust estimates of reproducibility and interpretable thresholds of change are necessary.

Reliability reflects the degree to which a measurement is consistent and reproducible when repeated under similar conditions. In functional tests, high reliability indicates that the results accurately reflect the subject’s performance and not measurement error, with the intraclass correlation coefficient (ICC) being the most common indicator used to quantify it (Koo and Li, 2016). For its part, the minimum detectable change (MDC) determines the smallest measurable change that can be considered real and not attributable to chance or measurement error; it is calculated from the standard error of measurement and the level of reliability and is expressed with a confidence level (usually 90% or 95%) (Lin, 1989; Haley and Fragala-Pinkham, 2006). In older populations or those with sarcopenia, knowing the MDC is particularly relevant, as small variations in physical performance can have a major impact on mobility, independence, and the risk of falls.

Therefore, the aim of the present study was to evaluate the test-retest measurement properties of the SARC-T in older adults diagnosed with sarcopenia according to the EWGSOP2 criteria, specifically its reliability, agreement, measurement error, and minimal detectable change at the 95% confidence level.

## 2 Methods

This was an observational methodological study with a test-retest design aimed at evaluating the measurement properties of the SARC-T, including reliability, agreement, measurement error and minimal detectable change on elderly subjects residing in nursing homes in the Community of Madrid between February and June 2023 and was approved by the ethics committee of Hospital Clínico San Carlos (Spain) with code: 23/010-E_TFM. All procedures were carried out in accordance with the Strengthening the Reporting of Observational studies in Epidemiology (STROBE) guidelines (Von Elm et al., 2008). The study was conducted in accordance with the principles described in the Declaration of Helsinki (WMA, 2024), and informed consent was obtained from all participants or their legal guardians prior to their voluntary participation in this research.

### 2.1 Participants

The sample was selected from among elderly subjects residing in nursing homes in the Community of Madrid.

The inclusion criteria were as follows: participants aged 65 years or older, able to stand unaided and with an adequate level of Spanish comprehension, who had been diagnosed with sarcopenia using the EWGSOP2 algorithm. The sample consisted of 55 institutionalized older adults with sarcopenia, including 31 women (56%) and 24 men (44%). Mean age was 85.3 ± 5.7 years (95% CI: 83.4–87.3; range: 73–95). Mean body weight was 61.3 ± 12.3 kg (95% CI: 57.1–65.6; range: 41–97.5), mean height was 154.8 ± 7.9 cm (95% CI: 152.1–157.5; range: 140–176), and mean body mass index was 25.6 ± 4.9 kg/m^2^ (95% CI: 23.5–26.8; range: 16.6–40.5) (Table 1).

**TABLE 1:** Age and anthropometric characteristics of participants.

Variable	Mean ± SD	(95% CI) Min-Max
Age	85.3 ± 5.7	(83.4–87.3) 73–95
Weight (kg)	61.3 ± 12.3	(57.1–65.6) 41–97.5
Height (cm)	154.8 ± 7.9	(152.1–157.5) 140–176
BMI (kg/m^2^)	25.6 ± 4.9	(23.5–26.8) 16.6–40.5
Women, n (%)	31 (56%)	​
Men, n (%)	24 (44%)	​

BMI: body mass index; CI: confidence interval.

The first step in identifying cases is to complete the SARC-F questionnaire; if the score is ≥ 4 points, muscle strength is assessed. To determine low muscle strength, the handgrip strength (HG) test is performed, confirming low muscle strength when the score is <27 kg in men and <16 kg in women. An alternative is to perform (5-STST), with a cut-off time of >15 s. If this is confirmed, probable sarcopenia is diagnosed. To confirm sarcopenia, muscle mass was assessed exclusively by bioelectrical impedance analysis (BIA) in all participants; DEXA was not used in the present study. The variable analysed is ASM, with cut-off points of 20 kg for men and 15 kg for women. Finally, physical tests are included to assess walking speed (GST) or the TUG test to determine the severity of the pathology.

The exclusion criteria were as follows: refusal to participate in the study, failure to sign the informed consent form, visual and/or hearing impairment that prevented understanding of the tests to be performed, consumption of psychoactive substances that affected the performance of the tests, and any orthopedic, neurological, and/or cardiorespiratory condition that prevented the participant from performing the various tests included in the study. Thus, cardiorespiratory conditions were considered only insofar as they precluded safe test execution; however, no specific analyses were planned according to cardiovascular disease status or the use of chronotropic and/or antihypertensive medication.

**FIGURE 1:**
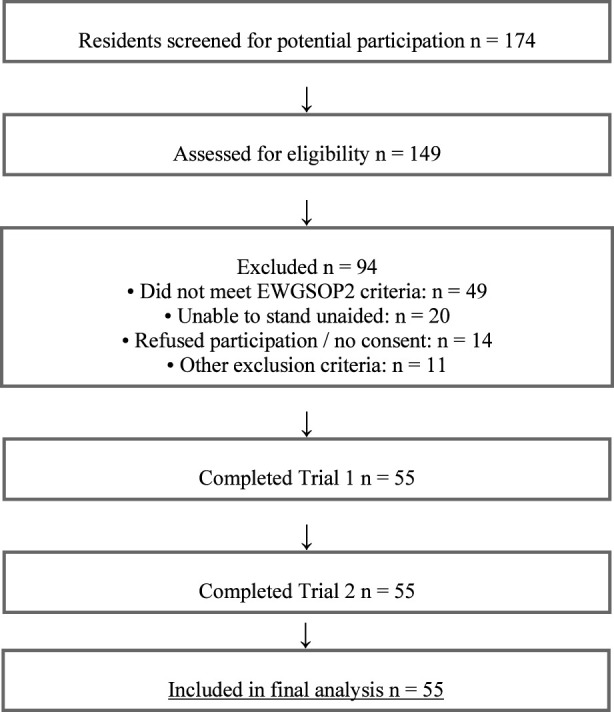
Process for selecting the participants included in the study.

Before formal data collection, participants completed two familiarization sessions conducted within the 5-day interval established in the protocol and under the same standardized conditions as the subsequent assessments. Two attempts were performed in each familiarization session. These sessions were intended to ensure proper understanding of the instructions, reduce erratic attempts, and promote a stable execution pattern before the formal test and retest sessions. Data from the familiarization sessions were not included in the reliability analyses because they were considered part of the standardization procedure rather than part of the formal test-retest assessment. After applying the inclusion criteria, the final sample for our study consisted of 55 subjects with sarcopenia (Figure 1).

### 2.2 Variables of study

The study variables were grouped into two domains.

Anthropometric variables: body weight and height were measured according to standardized procedures (WHO, 2019). Body mass index (BMI) was calculated as weight in kilograms divided by height in meters squared.

Performance variable: average propulsive velocity was measured using the SARC-T test.

### 2.3 Study procedures

This study used a repeated measures design to evaluate the test-retest reliability of a test based on the velocity of movement during the action of rising from a chair, measured using an optoelectronic encoder (Velowin v.1.7.232, Instrumentos y Tecnología Deportiva; Murcia, Spain). All tests were administered in two separate sessions, maintaining the same time window (±2 h) in order to control circadian rhythms (Pallarés et al., 2015) and under standardized environmental conditions (temperature between 18 °C–22 °C and relative humidity between 40%–55%). A 5-day interval was established between sessions to minimize possible learning effects and, simultaneously, avoid significant changes in the functional capacity of the participants. Before each session, body weight was recorded using a Detecto® scale (Lafayette Instruments Company, IN, USA; measurement range 0–150 kg; accuracy 200 g) and height was recorded using a Holtain® stadiometer (Holtain Limited; range 60–213 cm; accuracy 1 mm). All assessments in the present study were performed by the same evaluator, who was part of an evaluation team with prior training and experience in standardized test administration.

#### 2.3.1 SARC-T

To perform the SARC-T, each participant was asked to perform two consecutive repetitions from a 46-cm chair, without arm assistance, in order to improve reliability and reduce error. The starting position was upright standing, with knees and hips fully extended and feet shoulder-width apart. This standing start was selected not only to standardize the initial posture across participants, but also to facilitate stable optical detection of the reflective marker by the optoelectronic encoder. A reflective plate was placed over the participant’s right deltoid muscle, on the same side as the encoder, to ensure proper signal acquisition throughout the movement.

The protocol consisted of a controlled eccentric descent followed immediately by a maximal concentric rise. During the eccentric phase, participants were instructed to sit down at a controlled velocity (0.45–0.75 m/s), guided by real-time acoustic feedback provided by the linear velocity transducer software. Upon reaching the seated position and making full contact with the chair, participants were instructed to perform the concentric phase at maximum velocity until returning to the initial standing position. No intentional pause was allowed between the eccentric and concentric phases, and participants were instructed to perform the movement continuously.

The controlled eccentric phase was included as a standardized preparatory condition to homogenize the mechanical conditions preceding the concentric rise and to reduce variability associated with self-selected descent velocities, which may differ considerably in older adults. In addition, avoiding a pause between phases was intended to preserve the continuity of the movement, since pauses between eccentric and concentric actions may alter neuromuscular activation patterns, dissipate stored elastic energy, and modify movement efficiency. The selected eccentric velocity range was therefore used as an operational standardization criterion within the protocol and was not considered an outcome variable.

The velocity of execution was recorded using a validated and calibrated optoelectronic encoder (Velowin v.1.7.232, Instrumentos y Tecnología Deportiva; Murcia, Spain). The Velowin software automatically calculated mean propulsive velocity during the concentric phase using previously validated algorithms (Peña García-Orea et al., 2021). The primary outcome was defined as the best value obtained, since the SARC-T was conceived as a maximal performance test aimed at capturing the highest propulsive velocity achieved under standardized conditions. To assess the robustness of this methodological choice, an additional supplementary analysis was conducted using the mean of the two attempts from each session. In addition, within-session variability was quantified as the absolute percentage difference between both attempts.

### 2.4 Sample size

The sample size was calculated using the method proposed by Walter et al. (1998), as the planning objective was to test whether the reliability of the instrument exceeded a prespecified minimum acceptable ICC (ρ_0_ = 0.60), assuming an expected ICC of 0.80, with two measurements per participant (test and retest), α = 0.05, and 90% power. Under these assumptions, at least 52 evaluable participants were required. Allowing for a potential 10% loss, a minimum recruitment target of 57 participants was established. Although more recent approaches, such as Zou’s precision-based method, are also available, Walter’s method was considered appropriate for the hypothesis-testing design of the present study (Zou, 2012). Finally, 55 participants completed both evaluation sessions and were included in the analysis.

### 2.5 Statistical analysis

Statistical analysis was performed using IBM SPSS Statistics (v.30.0; IBM Corp., Armonk, NY, USA). Quantitative variables were described using mean ± standard deviation (SD) and 95% confidence interval (CI) when they were normally distributed; otherwise, they were reported as median [interquartile range]. Normality was assessed using the Shapiro-Wilk test and visual inspection (histograms and Q-Q plots). Systematic differences between the two sessions (test vs. retest) were analyzed using Student’s t-test for paired samples or, when normality was not met, using the Wilcoxon signed-rank test.

Test-retest reliability was estimated using ICC (3,1) (two-way mixed-effects model, absolute agreement, single measurement, reporting 95% CI), as the aim was to quantify reliability under the fixed and standardized conditions of the present protocol. The magnitude of reliability was interpreted according to standard criteria (poor <0.50; moderate 0.50–0.75; good 0.75–0.90; excellent >0.90) (Koo and Li, 2016). In addition, Lin’s coefficient of concordance (CCC) was calculated with its 95% CI to quantify concordance (Lin, 1989).

The SEM was estimated from the ICC using the classical formula SEM = SD·√(1−ICC), in accordance with the predefined analytical approach and to maintain consistency with the subsequent calculation of the MDC95, where SD corresponds to the standard deviation used for error estimation (e.g., combined SD of both measurements) (Weir, 2005). The minimum detectable change was calculated as MDC95 = SEM·1.96·√2 (Weir, 2005). The CIs for SEM and MDC were estimated by propagating the CI for ICC: SEM_inf = SD·√(1-ICC_sup) and SEM_sup = SD·√(1-ICC_inf), recalculating the corresponding MDC_inf and MDC_sup from these. In addition, a descriptive coefficient of variation (CV%) was calculated for each session to describe the relative within-session dispersion of performance values. Bland-Altman plots were used to assess the agreement between measurements (Weir, 2005). A linear mixed-effects model with session as a fixed effect and participant as a random intercept was added to formally assess systematic bias between measurements and to explore the relative contribution of inter- and intra-individual variability. This analysis was incorporated as a complement to the Bland-Altman and proportional bias analyses. Statistical significance was p < 0.05.

## 3 Results

The SARC-T showed high stability between sessions, with very similar mean values (0.29 ± 0.06 and 0.30 ± 0.07) and variability (21.7% and 22.8%), respectively, in both attempts. Test-retest reliability was high, as reflected by ICC and CCC values of 0.93. Furthermore, both with narrow confidence intervals. The measurement error was low, 0.017, and the MDC95 reduced, indicating that small variations in velocity can be interpreted as real changes in performance. These findings suggest that the SARC-T is an accurate and consistent tool for assessing the velocity of rising from a chair in people with sarcopenia (Table 2). As a supplementary analysis, test-retest reliability based on the mean of the two attempts per session showed good reliability, although lower than that observed for the primary analysis based on the best attempt (ICC = 0.789, 95% CI 0.670–0.861; CCC = 0.786, 95% CI 0.667–0.859). The corresponding SEM was 0.0226 m/s and the MDC95 was 0.0626 m/s (Supplementary Table S1). Within-session variability, expressed as the absolute percentage difference between both attempts, showed median values of 23.3% [IQR: 11.0–43.9] in session 1% and 26.4% [IQR: 12.9–40.7] in session 2 (Supplementary Table S2; Table 2).

**TABLE 2:** Test-retest reliability, agreement and measurement error of the SARC-T.

Inter-session test-retest reliability for the SARC-T (Trial 1 vs. Trial 2)				
Mean ± SD test 1 (m/s)	Mean ± SD test 2 (m/s)	Descriptive CV% within session 1	Descriptive CV% within session 2	ICC (CI 95%)
0.29 ± 0.06	0.30 ± 0.07	21.7%	22.8%	0.93 (0.88–0.96)
SEM (CI 95%)	MDC 95%	CI 95% MDC 95%	MDC%	CCC (CI 95%)
0.017 (0.013–0.023)	0.05	0.036–0.064	17%	0.93 (0.86–0.96)

SD: standard deviation; CV: coefficient of variation; ICC: intraclass correlation coefficient; CI: confidence interval; SEM: standard error of measurement; MDC: minimal detectable change; CCC: concordance correlation coefficient.

**FIGURE 2:**
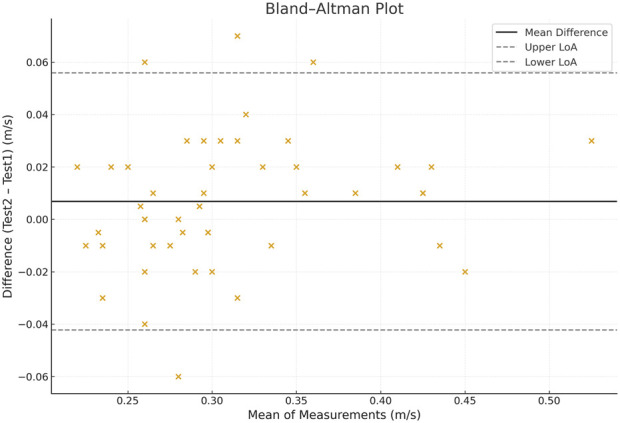
Bland-Altman plot.

Bland-Altman analysis showed a small inter-session mean bias of +0.008 m/s, indicating a limited systematic difference between measurements. The 95% limits of agreement ranged from −0.042 to +0.058 m/s. The mean bias was formally evaluated and showed a small but non-zero difference (p = 0.015). In addition, proportional bias was assessed by regressing the inter-session differences on the mean of both measurements, showing evidence of proportional bias (slope = 0.161; p = 0.009; R^2^ = 0.121) (Figure 2).

As a complementary analysis of systematic bias, a linear mixed-effects model was fitted with velocity as the dependent variable, session (Trial 1 vs. Trial 2) as a fixed effect, and participant as a random intercept. The model confirmed a small systematic session effect, with slightly higher values in Trial 2 than in Trial 1 (β = +0.008 m/s; 95% CI: 0.002 to 0.018; p = 0.012). Between-subject variance was greater than residual within-subject variance, suggesting that inter-individual heterogeneity accounted for an important part of the total observed variability.

## 4 Discussion

The main findings of this study indicate that the SARC-T demonstrates high test-retest reliability and low measurement error under standardized conditions in older adults with sarcopenia. However, these results should be interpreted in light of several methodological and interpretative factors that may influence the strength and clinical applicability of the findings.

Chair-rise performance has been widely used as an indicator of lower-limb function in older adults, and previous studies have shown its association with key features of sarcopenia, including muscle strength, functional capacity, and adverse health outcomes (Yoshiko et al., 2021; Ryu et al., 2022; Garcia-Aguirre et al., 2025a; 2025b). In addition, sit-to-stand-based assessments such as the 5-STST, GST, and TUG are commonly used in clinical and research settings (Bohannon et al., 2010; Bohannon, 2011; Muñoz-Bermejo et al., 2021; Ozsoy and Aksoy, 2024). Importantly, the SARC-T should not be interpreted as a conventional sit-to-stand test, as the protocol includes a controlled eccentric descent immediately followed by a maximal concentric rise. This eccentric phase was incorporated as a standardized preparatory condition to reduce variability associated with self-selected movement strategies and to ensure more homogeneous mechanical conditions across participants.

The assessments were performed at intervals of less than 7 days between each measurement, with the aim of minimizing the influence of possible physiological changes in muscle strength and, at the same time, avoiding learning effects derived from repetition of the test (Wang et al., 2009). This period has been described in the literature as adequate to ensure the stability of the participants’ physical condition without compromising the independence of the measurements.

The inter-session reliability analysis yielded high ICC and CCC values (0.93), indicating a high degree of consistency between repeated measurements (Landis and Koch, 1977). Nevertheless, reliability indices such as ICC primarily reflect relative consistency and do not necessarily imply absolute agreement between measurements (Koo and Li, 2016; Weir, 2005). In this regard, the Bland-Altman analysis provides complementary information that is essential for interpreting measurement agreement.

The Bland-Altman analysis revealed a small but statistically significant mean bias, together with evidence of proportional bias, indicating that agreement between sessions is not entirely uniform across the range of measured values. Although the magnitude of the mean bias was limited, its statistical significance, combined with the presence of proportional bias, suggests that measurement differences may vary depending on the level of performance. This finding has important implications, as it indicates that the SARC-T may behave differently across the spectrum of functional capacity, potentially affecting the interpretation of results at higher or lower performance levels. Therefore, while the overall level of agreement may be considered acceptable, it should not be assumed to be constant across all individuals or performance ranges.

The limits of agreement observed in this study, although relatively wide, should be interpreted within the context of the studied population and the inherent variability of functional performance in frail older adults. Given the frailty and functional heterogeneity of the studied population, a certain degree of variability and wider limits of agreement may be expected, which should be considered when interpreting repeated measurements in similar clinical contexts. From a clinical perspective, these limits reflect the expected range of variation between repeated measurements and highlight the importance of considering measurement error when interpreting changes over time. In this regard, the MDC95 provides a useful complementary metric, as it allows clinicians and researchers to determine whether observed changes are likely to exceed measurement-related variability (Haley and Fragala-Pinkham, 2006; Weir, 2005). However, the clinical acceptability of both the limits of agreement and the MDC95 remains uncertain and context-dependent and requires the establishment of clinically meaningful thresholds.

The measurement error estimated through SEM was low, supporting the precision of the instrument under the standardized conditions of the present protocol. However, the relatively high within-session variability (CV%) observed in both sessions indicates a moderate level of dispersion between repeated attempts within the same session. This variability should not be interpreted in isolation but rather in conjunction with other reproducibility indices. Importantly, it may have practical implications at the individual level, as it suggests that performance may fluctuate between attempts, potentially limiting the precision of single-measure assessments. Although this variability is likely influenced by the marked inter-individual heterogeneity characteristic of a frail population with sarcopenia, it should be acknowledged as a relevant source of uncertainty when interpreting individual performance.

From an instrumental perspective, previous validation studies of the optoelectronic system used in this study have reported substantially lower technical error, suggesting that the variability observed here is unlikely to be attributable solely to the measurement device (García-Ramos and Pérez-Castilla, 2018; Laza-Cagigas et al., 2019). This supports the interpretation that biological and functional variability plays a major role in the observed dispersion.

The supplementary analysis comparing the use of the best attempt versus the mean of two attempts showed lower reliability when the mean was used. This finding supports the use of the best-attempt criterion in maximal performance tests; however, it should be interpreted alongside the observed within-session variability, which indicates that occasional submaximal or inconsistent attempts may occur.

When compared with previous studies evaluating the reliability of functional tests in older adults, the ICC values observed in the present study are comparable to or slightly higher than those reported for the 5-STST, GST, and TUG (Ozsoy and Aksoy, 2024). This suggests that the SARC-T, when instrumented with an optoelectronic system, may enhance the objectivity of chair-rise assessment by quantifying movement velocity rather than relying exclusively on total execution time, although this potential advantage should be interpreted within the constraints of the present study design.

From a clinical and practical perspective, the applicability of the SARC-T should be interpreted with caution. Although the test provides an objective and instrumented measure of chair-rise performance, its reliance on optoelectronic equipment may limit its feasibility in routine clinical settings, particularly in low-resource environments. Consequently, its use may be more appropriate in specialized clinical or research settings where standardized conditions can be ensured.

Several limitations should be considered when interpreting the results of this study. First, the sample consisted of institutionalized older adults with sarcopenia from a single center, which may substantially limit both the precision of the estimates and the generalizability of the findings to other populations. Second, although the sample size met *a priori* requirements (Walter et al., 1998), it remains relatively modest. Third, performance in maximal tests may be influenced by factors such as motivation, fatigue, pain, and comorbidities (Wang et al., 2009). Fourth, the use of a fixed chair height may have introduced variability related to anthropometric differences. In addition, although the SEM was estimated using the conventional ICC-based approach and Walter’s method was appropriate for the hypothesis-testing aim of the present study, future research may complement these methods by exploring model-based SEM estimates and more recent precision-based approaches for ICC sample size determination, such as Zou’s method. Finally, the present study focused exclusively on test-retest reliability and did not assess other key measurement properties, such as inter-rater reliability, responsiveness to intervention, or external validation.

Taken together, these findings provide preliminary evidence supporting the reproducibility of the SARC-T under standardized conditions in a specific population of older adults with sarcopenia. However, the presence of systematic and proportional bias, the observed within-session variability, and the contextual limitations related to sample characteristics and feasibility highlight the need for cautious interpretation. Future research should evaluate additional measurement properties, explore its performance in more diverse populations, and establish clinically meaningful thresholds to support its potential application in clinical practice.

## 5 Conclusion

Under the standardized conditions of the present protocol, the SARC-T demonstrated high test-retest reliability and low measurement error in institutionalized older adults with sarcopenia. These findings provide preliminary but limited evidence supporting its reproducibility as an instrumented chair-rise measure in this specific population. However, the presence of statistically significant mean and proportional bias indicates that agreement between measurements is not entirely uniform across the range of performance, which may influence interpretation at the individual level. In addition, the relatively high within-session variability suggests that performance may fluctuate between attempts, supporting the need for cautious interpretation when relying on single measurements.

The MDC95 may serve as a useful reference to determine whether observed changes exceed measurement-related variability; however, its clinical interpretation should be considered alongside the limits of agreement and the absence of established clinically meaningful thresholds. From a practical perspective, the applicability of the SARC-T in routine clinical settings may be limited by the requirement for specialized optoelectronic equipment, and its use may be more appropriate in controlled or research environments.

Overall, while this study provides relevant evidence on the reproducibility of the SARC-T, further research is required to evaluate additional measurement properties, including inter-rater reliability, responsiveness to intervention, and external validation in more diverse populations, before supporting its broader use as a clinical monitoring tool.

## Data Availability

The raw data supporting the conclusions of this article will be made available upon request to the corresponding author.
