# Supplementary material for: Measurement properties of the SARC-T: test-retest reliability, agreement and minimal detectable change in older adults with sarcopenia
Source: Front Aging. 2026 May 29;7:1822206. doi: 10.3389/fragi.2026.1822206 (PMC13260494; doi:10.3389/fragi.2026.1822206)
Supplement: Supplementary file 1 [file Table1.docx]

**Supplementary Table S1.** Test-retest reliability of the SARC-T using the mean of the two attempts per session.

| **Session 1 mean ± SD** | **Session 2 mean ± SD** | **ICC (95% CI)** |
| --- | --- | --- |
| 0.28 ± 0.04 m/s | 0.29 ± 0.05 m/s | 0.79 (0.67-0.86) |
| **CCC (95% CI)** | **SEM (95% CI) m/s** | **MDC95 (95% CI) m/s** |
| 0.78 (0.66-0.8) | 0.022 (0.018-0.028) | 0.062 (0.050-0.078) |

SD: standard deviation; ICC: intraclass correlation coefficient; CI: confidence interval; SEM: standard error of measurement; MDC: minimal detectable change; CCC: Concordance Correlation Coefficient.
