# Supplementary material for: Measurement properties of the SARC-T: test-retest reliability, agreement and minimal detectable change in older adults with sarcopenia
Source: Front Aging. 2026 May 29;7:1822206. doi: 10.3389/fragi.2026.1822206 (PMC13260494; doi:10.3389/fragi.2026.1822206)
Supplement: Supplementary file 2 [file Table2.docx]

**Supplementary Table S2.** Within-session variability of the SARC-T.

| **APD Trial 1**, median [IQR]: 23.3 [11.0-43.9]% | **APD Trial 2**, median [IQR]: 26.4 [12.9-40.7]% |
| --- | --- |

APD: Absolute percentage difference.
